# Narrow-band imaging does not improve detection of colorectal polyps when compared to conventional colonoscopy: a randomized controlled trial and meta-analysis of published studies

**DOI:** 10.1186/1471-230X-11-100

**Published:** 2011-09-23

**Authors:** Luis C Sabbagh, Ludovic Reveiz, Diego Aponte, Sylvia de Aguiar

**Affiliations:** 1 Gastroenterology Department, Clínica Reina Sofía, Sanitas University Foundation, Bogota, Colombia; 2 Research Institute, Sanitas University Foundation, Bogota, Colombia; 3 Gastroenterology Department, Clínica Colombia, Bogota, Colombia; 4 General Practice Department, Clínica Reina Sofía, Bogota, Colombia

**Keywords:** Randomized controlled trial, colonoscopy, polyps, narrow-band imaging

## Abstract

**Background:**

A colonoscopy may frequently miss polyps and cancers. A number of techniques have emerged to improve visualization and to reduce the rate of adenoma miss.

**Methods:**

We conducted a randomized controlled trial (RCT) in two clinics of the Gastrointestinal Department of the Sanitas University Foundation in Bogota, Colombia. Eligible adult patients presenting for screening or diagnostic elective colonoscopy were randomlsy allocated to undergo conventional colonoscopy or narrow-band imaging (NBI) during instrument withdrawal by three experienced endoscopists. For the systematic review, studies were identified from the Cochrane Library, PUBMED and LILACS and assessed using the Cochrane risk of bias tool.

**Results:**

We enrolled a total of 482 patients (62.5% female), with a mean age of 58.33 years (SD 12.91); 241 into the intervention (NBI) colonoscopy and 241 into the conventional colonoscopy group. Most patients presented for diagnostic colonoscopy (75.3%). The overall rate of polyp detection was significantly higher in the conventional group compared to the NBI group (RR 0.75, 95%CI 0.60 to 0.96). However, no significant differences were found in the mean number of polyps (MD -0.1; 95%CI -0.25 to 0.05), and the mean number of adenomas (MD 0.04 95%CI -0.09 to 0.17). Meta-analysis of studies (regardless of indication) did not find any significant differences in the mean number of polyps (5 RCT, 2479 participants; WMD -0.07 95% CI -0.21 to 0.07; I2 68%), the mean number of adenomas (8 RCT, 3517 participants; WMD -0.08 95% CI -0.17; 0.01 to I2 62%) and the rate of patients with at least one adenoma (8 RCT, 3512 participants, RR 0.96 95% CI 0.88 to 1,04;I2 0%).

**Conclusion:**

NBI does not improve detection of colorectal polyps when compared to conventional colonoscopy (Australian New Zealand Clinical Trials Registry ACTRN12610000456055).

## Background

Screening for colorectal cancer using fecal occult blood testing, sigmoidoscopy, or colonoscopy is recommended in several countries in people above 50 years of age with an average risk and earlier in people with a strong family history or other risk factors [1-3]. Adenomatous polyps are deemed to be precursors of colorectal cancer. Some studies have shown that removal of polyps and postpolypectomy surveillance decreases the incidence of colorectal cancer [1,4-6].

Colonoscopy is considered to be the reference standard against which the sensitivity of other colorectal cancer screening tests is compared [1-3]. However, assessing the sensitivity and specificity of colonoscopy by comparing colonoscopy versus tandem colonoscopies, CT colonography and colonic specimens showed that colonoscopy may frequently miss polyps and cancers [7-9]. Meta-analysis of six studies [7] found that the miss rate for polyps of any size was 22% (95% CI: 19 to 26%). The study also reported that the adenoma miss rate was 2.1%, 13%, and 26% for polyp sizes of 10 mm and higher, 5-10 mm and 1-5 mm respectively. In another study, the diameter and the number of polyps (≥ 3) were independently associated with a lower polyp miss rate, whereas sessile or flat shape was significantly associated with a higher miss rate [8]. Simmons et al. analyzed 10,955 colonoscopies performed by 43 endoscopists and found that longer withdrawal time was also associated with higher polyp detection rate, particularly for smaller polyps [10]. Another study found that most advanced adenomas (74%) and cancers (95%) were detected during the insertion [11].

Diverse reasons for the miss rate have been suggested, including incomplete colonoscopy, the quality of bowel preparation, lesion characteristics (location, number, shape and size), the endoscopist's experience, the operator's insertion and the withdrawal technique [8-12].

A number of techniques have emerged to improve visualization and to reduce the adenoma miss rate [13]. The narrow-band imaging (NBI) technology in conventional video colonoscopes uses special filters to narrow a light source, eliminating red, enhancing structures and rendering vascular structures in black [14-18]. During the conduction of this research, a number of other studies comparing the NBI technique with conventional colonoscopy have been published [19-34].

The objective of this randomized controlled trial (RCT) was to evaluate the effectiveness of NBI during colonoscopy withdrawal compared to the conventional procedure in detecting polyps and adenomas. The objective of the systematic review was to identify and evaluate all RCT that assessed the effectiveness of diagnostic and screening conventional colonoscopy compared to NBI colonoscopy in detecting polyps and adenomas.

## Methods

This open-label randomized controlled trial (RCT) was conducted at the Gastrointestinal Department of two private clinics (Clinica Reina Sofia and Clinica Colombia), both tertiary care referral centers, during a nine-month study period. All consecutive adult patients presenting for screening or diagnostic colonoscopy for a variety of indications (e.g. positive fecal occult blood test, abdominal pain, post-polypectomy surveillance, diarrhea) were eligible for the RCT. Patients were excluded if they had known colonic neoplasia, inflammatory or another significant colonic disease (e.g. fulminant colitis, documented acute diverticulitis); if they had previously undergone colorectal surgery; if they had had a previous colonoscopy in the last 12 months before enrollment; if there was known familial adenomatous polyposis; if they were specifically presenting for polypectomy or emergency colonoscopy; if they were receiving anticoagulant medication; when adequate patient cooperation or consent could not be obtained; if the patient had a contraindication for the procedure; and in the cases of poor bowel preparation; active bleeding; or pregnancy. The trial protocol was approved by the Institutional Review Board of the Research Institute of the Medical School of the Sanitas University Foundation; written and informed consent was obtained from all the patients enrolled in the study. The study was registered in the Australian New Zealand Clinical Trials Registry ACTRN12610000456055.

### Assignment to interventions

During the first part of the procedure, the colonoscope was inserted through the rectum and advanced to the large intestine using conventional colonoscopy in both groups (we did not use chromoendoscopy during the process of conventional colonoscopy observation). Thereafter, patients were randomly assigned to colonoscope withdrawal using either conventional wide-angle or NBI wide-angle colonoscopy (Olympus Corp: Olympus 180, CF-Q180AL#2) in examinations conducted by a total of three experienced examiners (each with over 5,000 colonoscopies performed and more than 15 years of experience, including a minimum of two years of experience with NBI colonoscopy). The colonoscopies were performed using high definition monitors. Appropriate and complete bowel preparation before colonoscopy was ensured using four liters of polyethylene glycol lavage until clear rectal fluid was evacuated and a cleaning enema. We categorized the quality of bowel preparation into excellent, good, fair, poor or inadequate.

Randomization was performed in blocks of 4 and 6 using a random table. Once the caecum had been reached and appropriate bowel preparation confirmed, an opaque sealed envelope with sequential numbering was opened and participants were allocated to either NBI or conventional colonoscopy withdrawal of the instrument.

Polypectomies were performed in the same session during withdrawal when possible. Polyps were removed using snare polypectomy or forceps biopsy, depending on the size of the polyps.

### Outcome measures

#### Baseline Characteristics

The following demographic data and medical history information were obtained before randomization for every eligible patient: gender, age, weight, height, indication for colonoscopy, previous colonoscopy, date of last colonoscopy, previous polyp resection, and familial history of colorectal cancer.

#### Primary outcome measure

1. Mean number of detected polyps and adenomas

2. Total number of polyps

#### Secondary outcome measure

1. Polyp detection rate (at least one polyp per patient)

2. Polyp size

3. Location

4. Total number of adenomas smaller than 5 mm

5. Time before finding the first polyp.

6. Final histological result. Histological studies were performed on all removed polyps.

7. Adverse events

### Sample size determination

The primary endpoint for this study was the mean number of detected polyps. We assumed from institutional data that the mean number of polyps per patient in the control group would be 0.32, with a standard deviation (SD) of 0.31. A clinically significant increase in polyp detection using the NBI system was determined to be 25%. Using a two-tail alpha error of 0.05, and beta error of 0.20 (power > 80%), 240 patients in each arm would be required to detect a difference.

### Data management and analysis

The database, created in Excel, was double-checked and transferred to SPSS 15.0^©^. Categorical variables were compared using the chi-square test. To evaluate the continuous variables, Student's t-test was used; P < 0.05 was considered statistically significant. The measurement of the intervention effect for dichotomous outcomes was the risk ratio (RR). The measurement of the intervention effect for continuous outcomes was assessed by the mean difference (MD).

### Systematic review of the current evidence

During the conduction of this study, a number of other RCTs comparing the NBI technique with conventional colonoscopy were published. We performed an advanced search strategy of studies comparing the conventional colonoscopy to NBI to detect colorectal polyps/adenomas (appendix 1). Relevant RCTs were identified from the Cochrane Library (2009, Issue 4), PUBMED (1966-December 2009), LILACS (1982-December 2009) and Scirus (http://www.scirus.com; December 2009). We also scanned bibliographies of relevant studies for possible references to additional RCT. Two authors independently decided which trials fit the inclusion criteria. Eligible RCT were included regardless of the language of publication. Two reviewers independently extracted the relevant data using a pre-designed data extraction form and any disagreement was resolved by consensus with all authors. We extracted year of publication; patient population; number of patients (by intention to treat); sociodemographics; endoscopic, and histologic outcomes; and adverse effects. The main outcomes considered were the mean number of polyps, the mean number of adenomas and the rate of patients with at least one adenoma.

A risk of bias evaluation of each RCT was done following the Cochrane Collaboration's tool for the assessment of these features [35]. To estimate differences between treatments we calculated a weighted treatment effect across RCTs. We expressed the results as risk ratio (RR) with 95% confidence intervals (CI) for dichotomous outcomes and weighted mean difference (WMD) with 95% CI for continuous outcomes. We imputed conservative standard deviations where necessary using the p-value from an independent two-sample t-test [35]. For the pooled analysis, we calculated the I2 statistic, which describes the percentage of total variation across studies caused by heterogeneity [35].

## Results

### Randomized Controlled Trial

Patients were enrolled during a three month period at the Reina Sofia Clinic and during a 2 month period at the Clinica Colombia, between September 2008 and May 2009. We included a total of 482 patients (62.5% female), with a mean age of 58.33 years (SD 12.91); 241 into the intervention (NBI) colonoscopy and 241 into the conventional colonoscopy group. Patients were enrolled during a three month period at the Reina Sofia Clinic and during a 2 month period at the Clinica Colombia.

**Figure 1:**
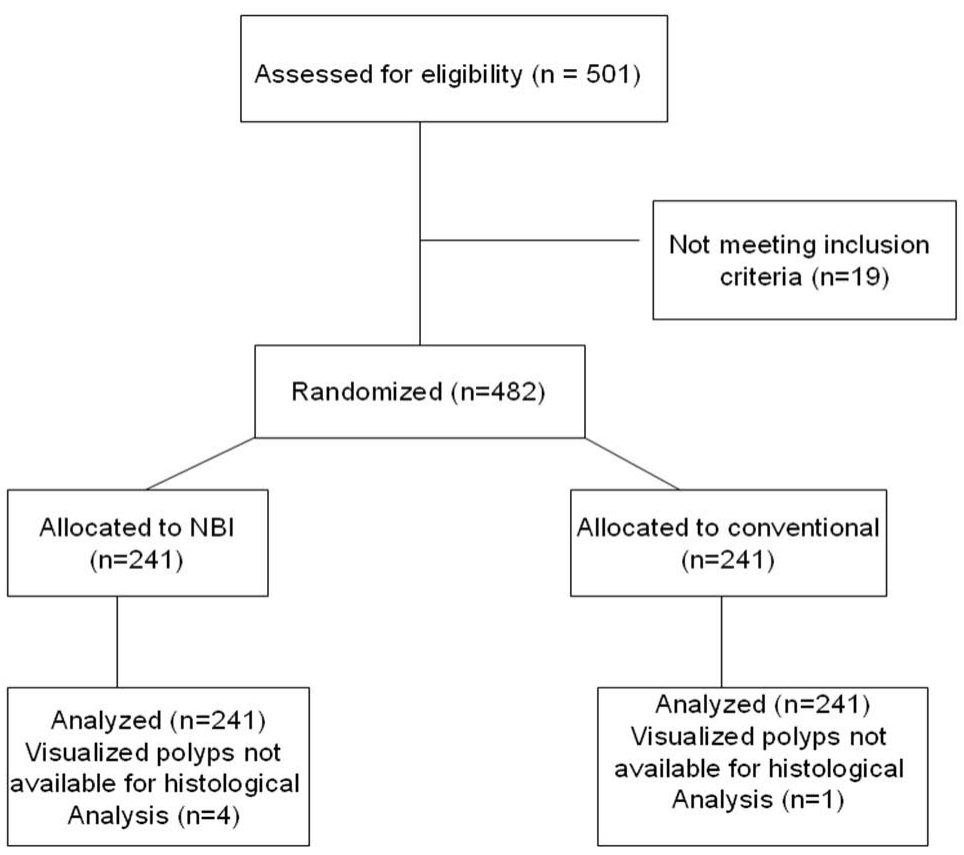
**Flow of participants through each stage of the randomized trial**.

The flow of participants through each stage of the randomized trial is described in Figure 1. Most polyps (67%) were found in the left colon; no significant difference was found between groups with regards to the location of the polyps. The total examination time did not differ significantly between the two groups (9.21 vs. 9.22; excluding polypectomy duration). Baseline characteristics of patients were similar among groups (Table 1).

**Table 1:** Baseline characteristics of patients and colonoscopy performance

Parameter	NBI group (n = 241)	Conventional group (n = 241)	Significance (p)
Age (years)	57.36 (SD 12.07)	59.29 (SD 12.91)	Ns

Gender M/F	86/155	95/146	Ns

Weight (kg)	66.73 (SD 12.99)	66.43 (SD 12.45)	Ns

Height (cm)	163.49 (SD 11.37)	163.11 (SD 8.57)	Ns

Total examination time (min)*	9.21 (SD 3.08)	9.22 (SD 3.58)	Ns

Previous colonoscopy	94 (39.0%)	98 (40.7%)	Ns

Indication:			

Screening	42 (17.4%)	37 (15.4%)	Ns

Surveillance	22 (9.1%)	18 (7.4%)	Ns

Diagnostic	177 (73.5%)	186 (77.2%)	Ns

Last colonoscopy (years)			
1 to 3	50	51	Ns
3 to 5	25	26	Ns
5 to 10	11	11	Ns
> 10	8	10	Ns

Previous polyp resection	26 (10.8%)	19 (7.9%)	Ns

Familial history of colorectal cancer	44 (18.3%)	36 (14.9%)	Ns

Excellent/good bowel preparation	167 (69.3%)	162 (67.5%)	Ns

*Not including polypectomy time.

The overall polyp detection rate per patient by visual inspection in the entire study group was 37.14% (77 polyps in the NBI group versus 102 in the conventional group). There was no protocol deviation, however 2.8% of visualized polyps were not available for histological analysis because polypectomy was delayed and no further procedure was performed until the termination of the study (Table 2).

**Table 2:** Main findings from the comparison between narrow-band imaging group and conventional group.

Outcome	NBI group (n = 241)	Conventional group (n = 241)	Measurement of the intervention effect*	Significance (p)
Total number of polyps (visual inspections)	77	102	RR 0.75 (0.60 to 0.96)	0.02

Proportion of patients with at least one polyp	50 (20.8%)	60 (24.9%)	RR 0.83 (0.60 to 1.16)	Ns

Mean number of polyps	0.32 (SD 0.73)	0.42 (SD 0.93)	MD -0.1 (-0.25 to 0.05)	Ns

Rate of polyp ≤ 5 mm	51 (21.2%)	62 (25.7%)	RR 0.82 (0.59 to 1.14)	Ns

Mean time to find the first polyp (seconds)	230.8 (SD 227.20)	220.81 (SD 233.93)	MD 10.80 (-30.27 to 51.87)	Ns

* Rate ratio (RR) and 95% CI or mean difference (MD) and 95% CI.

No significant difference was found in the mean number of polyps when comparing the conventional procedure to the NBI system (0.41 vs. 0.29). The overall detection rate of lesions (n = 174) and polyps (n = 169) by histological examination per patient in the entire study group were 36.1% and 35.1% respectively, with adenomas and hyperplastic polyps found, respectively, in 55.0% (n = 93/169) and 37.9% (n = 64/169) of all patients; tubulovillous and villous adenomas were found in 7,1% of polyps (Table 3). The overall rate of polyp detection was significantly higher in the conventional group compared to the NBI group (RR 0.75, 95% CI 0.60 to 0.96). Significant differences were also found in the rate of hyperplastic (RR 0.52, 95% CI 0.32 to 0.85; p = 0.009) and tubulovillous polyps (RR 0.11, 95% 0.01 to 0.87; p = 0.009). However, no significant differences were found in the mean number of polyps, the rate of polyps measuring less than 5 mm or the mean time to find the first polyp (Table 2).

**Table 3:** Histological characteristic of polyps detected in the narrow-band imaging group and conventional group on a per-polyp basis.

Outcomes (Number of polyps by histological category)	NBI group (n = 241)	Conventional group (n = 241)	Measurement of the intervention effect*	Significance (p)
Tubular adenoma or adenomatous polyp	46	47	0.98 (0.68 to 1.41)	Ns

Hyperplastic polyps	22	42	0.52 (0.32 to 0.85)	0.009

Villous adenoma	1	1	1.00 (0.06 to 15.90)	Ns

Tubulovillous adenoma	1	9	0.11 (0.01 to 0.87)	0.04

Adenocarcinoma	1	1	1.00 (0.06 to 15.90)	Ns

Other type of lesions	2	1	2 (0.18 to 21.91)	Ns

Total number of polyps	70	99	0.71 (0.55 to 0.91)	0.006

Total number of lesions	73	101	0.71 (0.57 to 0.92)	0.009

* Rate ratio (RR) and 95% CI or mean difference (MD) and 95% CI.

Of the adenomas, 9% were high-grade, with no significant difference between groups. Two adenocarcinomas were found (one in each group). There were no differences between the different examiners in the rate of detection of adenomatous and hyperplastic polyps. No serious adverse events were reported among groups during the procedure.

### Systematic Review of the current evidence

**Figure 2:**
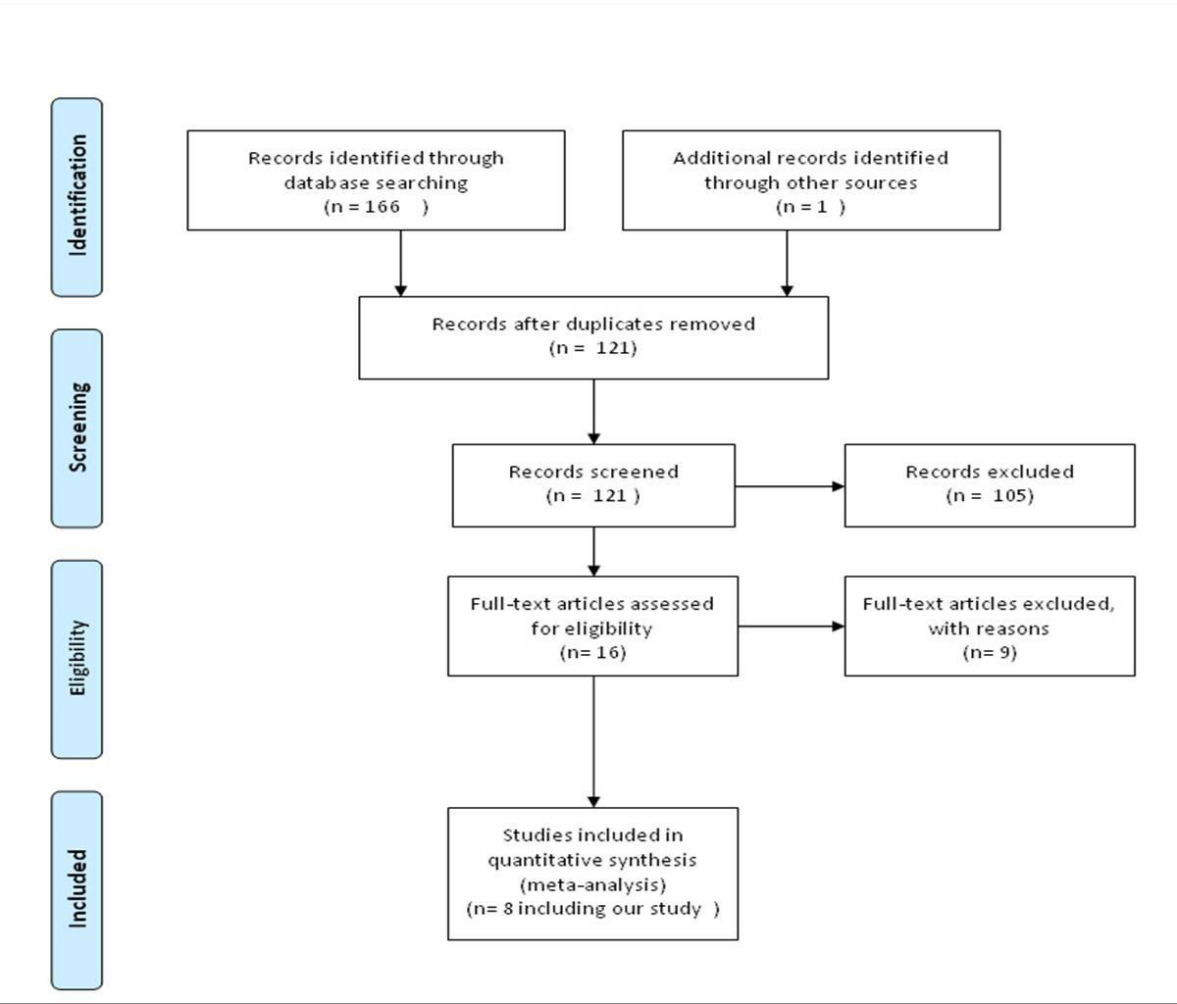
**Flow diagram of included and analysed randomized controlled trials**.

A total of 167 citations were identified from the diverse sources of information (Figure 2). Of the sixteen potentially RCTs screened [19-34], we excluded nine references because they were nonrandomized, they focused on predicting colon polyp histology, or they used other devices. Finally, seven RCTs meet the inclusion criteria [19-21,23,25-27] one of which was published as an abstract [27]. Characteristics of RCTs included in the meta-analysis are described in Table 4. One study was judged as having low risk of bias [21] and six RCTs were judged as having unclear risk of bias because the description of the method used to generate to conceal the allocation was unclear [19,20,23,27]; only one evaluator performed all the colonoscopies [25]; inadequate distribution of the NBI procedure among all participating endoscopists [26]; and a possible learning effect from the NBI during the initial phase of the trial [19]. Only in one RCT each patient underwent back-to-back colonoscopy [21]. The primary outcomes of interest were frequently reported incompletely and we had to impute standard deviations in four studies [19,21,26,27].

**Table 4:** Characteristics of included studies in the meta-analysis.

Study	Methods	Participants	Interventions	Primary Outcomes	Risk of bias*
Adler 2009	Open-label, prospective, randomized, controlled, multicenter trial	1256 patients, mean age, 64.4 years (range 31-87 y) undergoing screening colonoscopy	Wideangle colonoscopy using either conventional high-resolution imaging or NBI during instrument withdrawal (6 endoscopists). Exera II system.	Number of adenomas/number of patients examined	Unclear

Adler 2008	Open-label, prospective, randomized, controlled trial	401 patients with a mean age of 59.4 years (SD 13.4) undergoing diagnostic colonoscopy	Wideangle colonoscopy using either conventional high-resolution imaging or NBI during instrument withdrawal (7 endoscopists). Exera II system.	Adenoma detection rate	Unclear

East 2009 (abstract)	Open-label, prospective, randomized, controlled trial	214 high risk adenomas patients with a median age of 65 and 66 years in the NBI and conventional group respectively	Examination with NBI or white light (WLE), with high definition (HDTV) colonoscopes (3 endoscopists). Lucera system.	The number of patients with at least one adenoma detected.	Unclear

Inoue 2008	Open-label, prospective, randomized, controlled trial	243 patients (NBI mean age, 61.1 SD 13.5; conventional mean age 62.9 SD 11.3) undergoing surveillance or diagnostic colonoscopy	Colonoscopy using either conventional high-resolution imaging or NBI during instrument withdrawal (6 endoscopists). Lucera system.	Mean number of adenomas per patient	Unclear

Kaltenbach 2008	Open-label, prospective, randomized, controlled trial	276 patients with a mean age of 64 (SD 10) years (range 31 to 89), undergoing screening, surveillance or diagnostic colonoscopy	Wideangle colonoscopy using either conventional high-resolution imaging or NBI (6 endoscopists); back-to-back colonoscopy by the same endoscopist. Exera II system.	Neoplasm miss rate.	Low

Paggi 2009	Open-label, prospective, randomized, controlled trial	211 patients (from 50 to 69 years) with positive immunologic fecal occult blood tests	Colonoscopy withdrawal in white light versus NBI (6 endoscopists). Exera II system.	The detection rate of adenoma	Unclear

Rex 2007	Open-label, prospective, randomized, controlled trial	434 patients aged 50 years or older undergoing screening or surveillance colonoscopy	Colonoscopy withdrawal in white light versus NBI (only one endoscopist; high definition monitors were used). Exera II system.	Number of adenomas	Unclear

Sabbagh 2011	Open-label, prospective, randomized, controlled, multicenter trial	482 patients with a mean age of 58.3 (SD 12.9) undergoing screening, surveillance or diagnostic colonoscopy	Wideangle colonoscopy using either conventional high-resolution imaging or NBI (3 endoscopists). Exera II system.	Mean number of adenomas	

*The judgment for each entry involves answering a question, with answers "Yes" indicating low risk of bias, "No" indicating high risk of bias, and "Unclear" indicating either lack of information or uncertainty over the potential for bias

**Figure 3:**
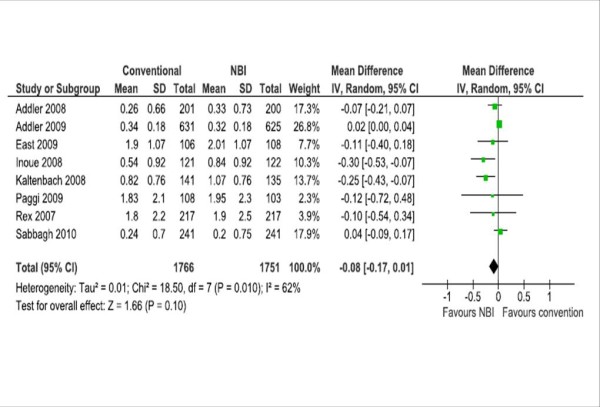
**Meta-analyses of the mean number of adenomas**.

**Figure 4:**
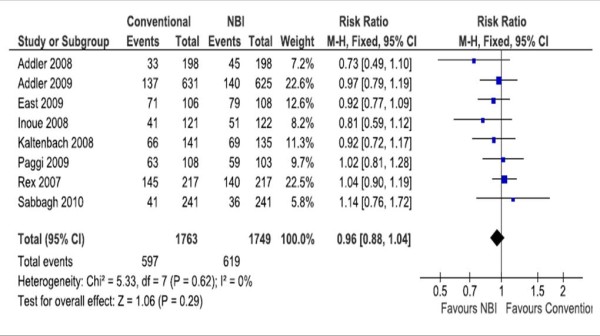
**Meta-analyses of the rates of patients with at least one adenoma**.

A meta-analysis of studies (including diagnostic, surveillance and/or screening colonoscopies) is showed in Table 5. No significant differences were found among groups in the mean number of polyps, the mean number of adenomas (Figure 3), and the rates of patients with al least one polyp or one adenoma (Figure 4). We performed a sensitive analysis comparing those studies that used Lucera or Exera II systems. Significant differences favoring the NBI system in the mean number of polyps (2 RCT, 457 participants; WMD -0.39 95% CI -0.62 to - 0.16; I2 0%) and the mean number of adenomas (2 RCT, 457 participants; WMD -0.22 95% CI -0.41 to - 0.04; I2 2%) were found when pooling data from RCTs that used the Lucera system [26,27].

**Table 5:** Meta-analysis of studies comparing conventional colonoscopy to NBI system.

Study (Year)	Outcome	Conventional colonoscopy (N)	NBI system (N)	RR* WMD** (95%CI)	Heterogeneity (I2) §
Adler 2009 East 2009 Inoue 2008 Kaltenbach 2008 Sabbagh 2011	Mean number of polyps	1241	1238	WMD -0.07 (-0.21 to 0.07)	68%

Adler 2009 Adler 2008 East 2009 Inoue 2008 Kaltenbach 2008 Paggi 2009 Rex 2007 Sabbagh 2011	Mean number of adenomas	1768	1749	WMD -0.08 (-0.17 to 0.01)	62%

Adler 2009 Adler 2008 Inoue 2008 Kaltenbach 2008 Sabbagh 2011	Rate of patients with al least one polyp	1336	1330	RR 0.90 (0.73 to1.11)	70%

Adler 2009 Adler 2008 East 2009 Inoue 2008 Kaltenbach 2008 Paggi 2009 Rex 2007 Sabbagh 2011	Rate of patients with at least one adenoma	1763	1749	RR 0.96 (0,88 to 1.04)	0%

Adler 2009 Adler 2008 Paggi 2009 Sabbagh 2011	Rate of patients with carcinoma	1181	1169	RR 1.20 (0.58 to 2.47)	0%

*Relative Risk and 95% confidence intervals (CI) for dichotomous outcomes were calculated by the Mantel-Haenszel fixed-effects model when I2 < 50%. Relative Risk and 95% confidence intervals (CI) for dichotomous outcomes were calculated by the Mantel-Haenszel random-effects model when I2 > 50%.

**Mean difference and 95% confidence intervals (CI) for continuous outcomes were calculated using the inverse variance and the fixed-effects model when I2 < 50%. Mean difference and 95% confidence intervals (CI) for continuous outcomes were calculated using the inverse variance and the random-effects model when I2 > 50%.

§ Low, moderate, and high levels of heterogeneity approximately correspond to I2 values of 25%, 50% and 75%, respectively [35].

No serious adverse events were reported. Taken into account that we only found eight RCTs, funnel plots for assessing publication bias was not performed.

## Discussion

### Main findings

According to our findings, the polyp detection rate per patient by visual inspection was significantly higher in the conventional colonoscopy group compared to the NBI group. In addition, significant differences favoring the conventional colonoscopy group were also found for some types of histological examination polyps (hyperplastic polyps and tubulovillous adenoma). However, the adenoma detection rate was similar in both groups.

Although meta-analysis of RCTs showed no significant difference for pre-specified primary outcomes, individual studies reported diverse findings [19-28]. Statistical heterogeneity may be explained by difference in the prevalence of polyps and adenomas in the population, the indication for colonoscopy (screening, surveillance and/or diagnostic), the age of the included population, and the examiner's experience among others. Findings of our review update those of a previous systematic review that included three RCTs concerning the detection of colorectal adenomas [36].

Only two RCTs found a significant difference in the mean rate of adenomas favouring the NBI group [21,26]. One RCT, which included 243 patients, found a significant difference in the rate of adenoma detection favoring the NBI group (22% vs. 14%), including the subgroups of patients having polyps measuring less than 5 mm. The authors of the study recommended the routine use of the NBI system for surveillance of diminutive adenomas [26]. Another study [21] found that the NBI system significantly increased the total number of adenomas detected as well as the number of diminutive adenomas in the distal colon [21]; however the rate of missed lesions between the NBI and conventional group was similar. Another RCT found that the number of diminutive (< 5 mm) adenomas was significantly higher in the NBI group [26]. One RCT reported a significantly higher detection in the mean number of flat adenomas in the NBI group [27], one study reported the opposite [20] and two RCTs did not find any significant difference [23,25].

In our study, colonoscopies were performed by experienced examiners in both techniques and included diagnostic and screening colonoscopies from two different institutions. Adenoma rates in larger colonoscopy trials vary widely. The overall detection rate of polyps and adenomas by histological examination were 35.1% and 22% respectively, which is similar to the rates reported in other studies [20,37-40]. However some RCTs found higher rates of polyps and adenomas; the difference can be explained by heterogeneous included population. The lower detection rate in our study may be due to the withdrawal time but also to the lower prevalence in our population. A number of published studies have evaluated the prevalence of polyps and adenomas in Colombia. Overall, the prevalence of colonic adenomas is lower when compared with rates reported in other regions [41-44]. In addition, around one third of patients in our RCT had already had a colonoscopy. Although differences in polyp frequency between screening and diagnostic colonoscopy have been reported, some studies have found similar rates [40]. Additionally, no differences were found in primary outcomes between colonoscopies performed in the two different clinics, or when different periods of time were compared (first 241 colonoscopies vs. last 241 colonoscopies).

### Limitations of the study

The findings of this RCT have some limitations, mostly due to the lack of tandem colonoscopy in both groups. Difference in the overall rate of polyps could have been due to selection bias (patients with more polyps could have been included in the conventional group by chance). However, we found no significant differences among participants in the baseline characteristics and the colonoscopy performance; we included a significant number of patients and we concealed the allocation of patients to minimize any possible bias. Our data shows that the white-light group had 20.8% higher detection rate of adenomas than did the NBI group. As both groups had similar withdrawal time, the white-light group could have had better mucosal visualization during the withdrawal phase compared to that of the NBI group (the darkening of the image associated with the use of NBI). This may have lead to the finding of significantly greater number of polyps found in the white-light group.

In addition, the fact that bowel preparation was not excellent in one third of patients may have contributed to the poorer performance of the NBI visualization. As screening colonoscopy is not usually recommended in our country in people above 50 years of age with average risk, we had an important proportion of diagnostic colonoscopies.

Concerning the review, we pooled data from studies that included heterogeneous populations and indications. The use of varied endoscopic systems as well as differences in colon preparation of participants between studies may have had some impact on findings. Uraoka et al noticed that significant differences in the detection of adenomas where related to the type of endoscopic video system (either the sequential LUCERA series or the simultaneous EXERA-II series). They found that most positive studies used the LUCERA system while all of the negative studies used the EXERA-II system [45]. Although pooled estimates from two RCTs [26,27] support the use of LUCERA series, both studies where judged as having unclear risk of bias. More research is still needed to determine the efficacy of different NBI system settings for screening and surveillance colonoscopies, particularly to enhance the detection rate for flat adenomatous lesions [45].

Finally, there were differences in the report of flat or non-polypoid type neoplasm among studies which did not permit further pooled analysis.

## Conclusion

This RCT in two homogeneous practices did not show any objective advantage of the NBI technique over the conventional colonoscopy in terms of improved adenoma detection rate. Pooled estimated of published RCT showed no benefits of the NBI system over the conventional colonoscopy in terms of the mean number of polyps and the mean number of adenomas identified.

## Declaration of competing interests

The authors declare that they have no competing interests.

## Authors' contributions

LCS and DA conceived the study and participated in the study coordination. All authors participated in the study design, data analysis and contributed to preparing the manuscript, and read and approved the final manuscript.

## Appendix 1. Search strategy for PUBMED

((randomized controlled trial [pt] OR controlled clinical trial [pt] OR randomized [tiab] OR placebo [tiab] OR drug therapy [sh] OR randomly [tiab] OR trial [tiab] OR groups [tiab]) NOT (animals [mh] NOT (humans [mh] AND animals [mh]))) AND (Colonoscopy [mh] OR colonosco* [tw] OR (intestin* endoscop* [tw])) AND (narrow band [tw] OR NBI [tw])

## Pre-publication history

The pre-publication history for this paper can be accessed here:

http://www.biomedcentral.com/1471-230X/11/100/prepub
